# Semi-Automated Hydrophobic Interaction Chromatography Column Scouting Used in the Two-Step Purification of Recombinant Green Fluorescent Protein

**DOI:** 10.1371/journal.pone.0108611

**Published:** 2014-09-25

**Authors:** Orrin J. Stone, Kelly M. Biette, Patrick J. M. Murphy

**Affiliations:** The Interdisciplinary Health Sciences Research Laboratory, Colleges of Nursing and Science & Engineering, Seattle University, Seattle, Washington, United States of America; University of Edinburgh, United Kingdom

## Abstract

**Background:**

Hydrophobic interaction chromatography (HIC) most commonly requires experimental determination (i.e., scouting) in order to select an optimal chromatographic medium for purifying a given target protein. Neither a two-step purification of untagged green fluorescent protein (GFP) from crude bacterial lysate using sequential HIC and size exclusion chromatography (SEC), nor HIC column scouting elution profiles of GFP, have been previously reported.

**Methods and Results:**

Bacterial lysate expressing recombinant GFP was sequentially adsorbed to commercially available HIC columns containing butyl, octyl, and phenyl-based HIC ligands coupled to matrices of varying bead size. The lysate was fractionated using a linear ammonium phosphate salt gradient at constant pH. Collected HIC eluate fractions containing retained GFP were then pooled and further purified using high-resolution preparative SEC. Significant differences in presumptive GFP elution profiles were observed using in-line absorption spectrophotometry (A_395_) and post-run fluorimetry. SDS-PAGE and western blot demonstrated that fluorometric detection was the more accurate indicator of GFP elution in both HIC and SEC purification steps. Comparison of composite HIC column scouting data indicated that a phenyl ligand coupled to a 34 µm matrix produced the highest degree of target protein capture and separation.

**Conclusions:**

Conducting two-step protein purification using the preferred HIC medium followed by SEC resulted in a final, concentrated product with >98% protein purity. In-line absorbance spectrophotometry was not as precise of an indicator of GFP elution as post-run fluorimetry. These findings demonstrate the importance of utilizing a combination of detection methods when evaluating purification strategies. GFP is a well-characterized model protein, used heavily in educational settings and by researchers with limited protein purification experience, and the data and strategies presented here may aid in development other of HIC-compatible protein purification schemes.

## Introduction

Hydrophobic interaction chromatography (HIC) is a well-established technique that separates a recombinant or endogenous protein of interest from crude cellular lysate based on differences in protein surface hydrophobicity. It is often chosen as a purification method for isolating proteins that lack an affinity tag or when other chromatographic methods such as ion exchange chromatography (IEX) or size exclusion chromatography (SEC) fail to provide adequate resolution [1]. In a multi-step protein purification, HIC is often used as an initial ‘capture’ step and then followed by one or more ‘polishing’ steps utilizing IEX or SEC [2]. Proteins best suited for HIC purification include those containing one or more defined hydrophobic surface regions that are able to withstand exposure to high salt concentrations [3], [4].

In contrast to other liquid chromatography-based methods, HIC commonly requires experimental determination (i.e., scouting) in order to select an optimal chromatographic medium or buffer conditions for isolating a particular HIC-compatible protein of interest [3]. Commercially available HIC media differ in functional group chemical structures, hydrophobicities, and densities, as well as size of the inert matrix beads to which the functional groups are attached. These differences in HIC media combine to produce unique chromatographic elution profiles. Examples of common functional groups covalently bound to HIC matrix beads include –O–(CH_2_)_3_CH_3_ (butyl), –S–(CH_2_)_3_CH_3_ (butyl-S), –O–(CH_2_)_7_CH_3_ (octyl), and an aromatic –O–C_6_H_5_ (phenyl). Functional group densities for specific HIC media typically range from 5–50 µmol/ml of medium, and matrix bead diameters range from 30–100 µm. Smaller bead diameters and higher functional group densities tend to provide greater chromatographic resolution, whereas larger bead diameters and lower functional group densities are preferred for separations of more concentrated samples and operation at faster flow rates. Additional variables commonly altered to improve HIC protein purification elution parameters include elution buffer molarity, pH, and chromatographic flow rate [3], [4], [5].

While it is possible to compare HIC media and buffer conditions manually, modern chromatography systems include programmable scouting functions that allow for sequential testing of different HIC columns and buffer conditions with limited operator intervention. Automated column scouting allows for immediate repetition of a purification protocol using different HIC media [2]. Since each HIC medium is contained within a pre-plumbed chromatography column with controlled access through column selection valving and dynamic sample loop injection, automated column scouting allows for increased reproducibility between chromatography runs and a more streamlined workflow [6]. In-line eluate analysis further increases purification efficiency by detecting proteins of interest using characteristic UV-visible light absorption spectra during the chromatography run. Once in-line analyses have been confirmed for the protein of interest using post-run techniques (e.g., SDS-PAGE, western blotting, and Bradford assays), it is often possible to monitor the presumptive target protein elution based on in-line detection methods alone, which reduces associated time and costs [3], [4].

Green fluorescent protein (GFP) is a highly stable and well-characterized protein with multiple hydrophobic surface moieties, making it particularly suitable for HIC methods development and chromatography training purposes. GFP has been especially useful as a model protein because of its stability, efficient expression in *E. coli*, mutability, and unique emission of green light fluorescence when excited by UV light [7], [8], [9]. GFP has a well-documented absorption (i.e., excitation) maximum of 395 nm and emission maximum of 509 nm, which can be readily detected by UV-visible light absorption spectrophotometry and fluorimetry, respectively [7], [8], [9]. Although recombinant GFP can be mutagenized to contain an affinity label, such as a poly-histidine tag, the purification of GFP using chromatographic techniques other than affinity chromatography has become a common instructional tool. It is regularly used in training modules and undergraduate biochemistry laboratories to increase student familiarity with fundamental principles of protein purification, instrumentation, and techniques [10], [11]. Additionally, HIC may allow for purification of GFP-fusion proteins that cannot be readily purified by GFP-directed affinity chromatography [12].

Although the chromatographic purification of untagged GFP has been described in varying levels of detail utilizing several methods, neither the use of two-step HIC-SEC for GFP purification nor HIC column scouting elution profiles of GFP have been reported previously. Examples of previously reported GFP purification approaches include affinity purification with monoclonal antibodies [13], sequential HPLC-based SEC-IEC [14], preparative PAGE [15], and HIC [16], [17], [18]. While these previously described techniques result in highly purified GFP, each contains elements that may limit its adaptability to other HIC-compatible protein purifications. Examples of potential limitations of these previously described methods include exposure to organic solvents (e.g., three-phase partitioning [18]), heat (e.g., 60–72°C incubation [16], [17]), electro-elution [15], high pressure [14], and GFP antigen recognition [13]. There are few examples in the literature providing direct comparisons of untagged GFP purification elution profiles or eluate analyses following HIC column scouting. Presenting chromatography elution profile data is beneficial, as they provide a fuller assessment of HIC medium separation and can guide subsequent target protein purification studies. Identifying preferred HIC media to be used for target protein purification allows other investigators to reduce substantially or eliminate fully the time and costs associated with HIC column scouting. Target protein purity and yield data can be used as benchmarks for comparison by other investigators, including those with limited experience in liquid chromatography and those using HIC-based protein purification as an educational laboratory experience.

While HIC generally necessitates empirical determination of optimal media to use for each new protein purification, it is possible to develop a general column scouting protocol to ascertain how various HIC media compare. The purpose of this study was to assess the utilization of an efficient semi-automated column scouting method to determine a preferred HIC medium for GFP capture, and to use this technique as the first step in a two-step GFP purification strategy. We observed unexpected dissimilarities in in-line A_395_ absorbance detection and post-run 509 nm fluorimetry data, which resulted in differences in presumptive GFP elution profiles and were resolved by SDS-PAGE and western blot analysis. By combining the initial capture of GFP using the preferred scouted HIC medium with a high resolution preparative SEC polishing step, we were able to obtain a concentrated product of GFP possessing >98% purity and approximately 50% yield.

## Materials and Methods

### Liquid Chromatography System

All chromatography was completed using a BioLogic DuoFlow Pathfinder 20 (Bio-Rad) medium pressure liquid chromatography system containing a Maximizer automated buffer blending unit, DynaLoop dynamic sample loading loop, AVR7-3 injection valve, automated AVR9-8 column selection valves, QuadTec multiple wavelength detector, UV detector, and dual BioFrac eluate fraction collectors, as illustrated in Figure S1. Eluate was collected continuously throughout all chromatography runs. A splitter valve connected to dual fraction collectors was used to divert 100 µl of eluate from each 1 ml fraction into 96 well microtiter plates for post-run analysis while the remaining 900 µl of each fraction was collected in 12×100 mm borosilicate test tubes for subsequent purification steps. The multiple wavelength and UV detectors recorded in-line light absorbance at 280 nm and 395 nm throughout the run, which provided an approximate indicator of total protein elution and GFP elution, respectively. In-line chromatography system pressure, conductivity, and pH monitors were used to confirm stable buffer conditions throughout the chromatography runs. The presence of GFP in loaded samples and fractionated eluate was confirmed by visual inspection under UV light. All chromatography instrumentation was housed and operated in a dedicated 4°C cold room to minimize protein degradation.

### Bacterial Expression and Lysate Preparation

GFP was produced in *E. coli* transfected with the pGLO cycle 3 variant GFP expression plasmid (Bio-Rad) following the manufacturer’s instructions. Briefly, an Erlenmeyer flask containing 1 L of Luria broth supplemented with ampicillin (100 µg/ml) and arabinose (6 mg/ml) was inoculated with starter culture and incubated at 32°C overnight. The culture was pelleted by centrifugation at 3,250×*g* for 5 min and resuspended in 50% (v/v) TE buffer (10 mM Tris, 1 mM EDTA, pH 8.0) containing 1 mg/ml lysozyme. The resultant slurry was sonicated (50% duty cycle, 5 min) on ice, centrifuged at 3,250×*g* for 5 min, and clarified by 0.45 µm syringe filtration. Clarified lysate was aliquoted, flash frozen, and stored at −20°C. Throughout subsequent purification steps, clarified lysate and fractionated HIC-SEC eluate were maintained on ice or at 4°C.

### Column Scouting and Target Protein Isolation

Clarified bacterial lysate containing overexpressed GFP was diluted 50% (v/v) in 2.4 M ammonium sulfate, pH 6.8, and loaded into the dynamic sample loop of the pre-plumbed chromatography system, as described previously [6]. Dynamic sample loop loading, sample injection, column washing, and protein elution occurred using a constant flow rate of 1 ml/min. Immediately prior to each HIC column scouting run, 1 ml of lysate was injected from the dynamic sample loop and adsorbed onto a 1 ml HIC HiTrap Sepharose scouting column (HIC Selection Kit, GE Life Sciences). Scouting columns contained butyl, butyl-S, octyl, and phenyl ligands with purported particle diameters ranging from 34–90 µm and ligand densities ranging from 5–50 µmol/ml. Following preliminary scouting runs, the four HIC columns with most distinct GFP elution characteristics were selected for in-depth analysis. These four GE Life Sciences columns were: Butyl Sepharose High Performance (34 µm matrix, 50 µmol/ml ligand density); Octyl Sepharose 4 Fast Flow (90 µm matrix, 5 µmol/ml ligand density); Phenyl Sepharose High Performance (34 µm matrix, 25 µmol/ml ligand density), referred to as phenyl-34; and, Phenyl Sepharose 6 Fast Flow High Substitution (90 µm matrix, 40 µmol/ml ligand density), referred to as phenyl-90.

GFP elution from each HIC scouting column occurred over a 12 ml linear gradient of 2.4 M ammonium sulfate (100% Buffer B) to 0 M ammonium sulfate (0% Buffer B) contained within a 0.2 M sodium phosphate buffer system at a constant pH of 6.8. Each HIC column was equilibrated with 2 ml of 100% Buffer B, injected with 1 ml sample, and washed with 5 ml of 100% Buffer B prior to the linear gradient elution. Following elution, the column was washed with 7 ml of 0% Buffer B and then re-equilibrated in 100% Buffer B. Each column scouting run was completed in its entirety before the next scouting began, and total HIC column scouting run volumes were 32 ml/column scouted. All scouting runs were performed immediately in succession under the control of automated sample injection and column selection valving without disruption to the backpressure or flow rate. One revision from our previously reported HIC scouting method [6] was that the present study included an additional 2 ml of 0% Buffer B isocratic flow at the end of each scouting run in order to ensure tightly bound proteins were fully removed from the column prior to initiating the next run.

Eluate fractions from the HIC scouting runs that were identified as containing retained GFP were pooled and concentrated via centrifugal filtration using Ultra-15 centrifugal filters with a 10 kDa molecular weight cutoff (Amicon) at 4,000×*g*. Concentrated fractions from each column were reconstituted to the initial 1 ml load volume in HE buffer (10 mM Hepes, 1 mM EDTA, pH 7.4). The target protein was further purified by high resolution preparative SEC using a 16×600 mm Superdex 200 column (GE Life Sciences) with a total bed volume of 120 ml. Gel filtration standards (Bio-Rad) were used to approximate molecular weights of corresponding SEC eluate fractions. The concentrated GFP-containing HIC eluate from the preferred HIC column was loaded into a 1 ml static sample loop, injected onto the SEC column, and fractionated into 150 ml of total elution volume at a flow rate of 0.5 ml/min in HE buffer. GFP-containing SEC eluate fractions were identified by in-line and post-run analyses, pooled, and re-concentrated to a final volume of 1 ml by centrifugal filtration, as described above.

### Post-run Eluate Analysis and Target Protein Quantification

Aliquots of HIC and SEC eluate were spectrophotometrically assayed for total protein and GFP using Bradford and fluorimetry assays, respectively. Total protein content in each HIC eluate fraction was determined by performing a Bradford assay (Bio-Rad) on 15 µl of undiluted eluate according to the manufacturer’s instructions. GFP-associated fluorescence was measured in 50 µl of each HIC and SEC eluate fraction with a Synergy HT multi-mode microplate reader (BioTek) using excitation and emission wavelengths of 395 nm and 509 nm, respectively.

Molecular weights and relative amount of protein in HIC and SEC fractions were determined Coomassie staining and western blot. Samples were boiled in Laemmli sample buffer (Bio-Rad) under denaturing conditions, electrophoresed on 12% polyacrylamide gels containing SDS, and transferred to Immobilon-P PVDF membranes (Millipore). Membranes were stained with Coomassie brilliant blue (Sigma), and molecular weights were compared against broad range SDS-PAGE molecular weight protein standards (Sigma).

For western blotting, PVDF membranes containing duplicate samples to those used for Coomassie staining were incubated for at least 15 min in blocking buffer (TBS with 0.5% Tween 20 and 2.5% BSA) and then probed with an ABfinity recombinant rabbit monoclonal anti-GFP primary antibody (Molecular Probes) at a dilution of 1∶1000 in blocking buffer for 3 h at room temperature. Membranes were washed with TBS and incubated with a goat anti-rabbit horseradish peroxidase-conjugated secondary antibody (Pierce) at a dilution of 1∶500. Following washing in TBS, immune-specific GFP protein bands were visualized using ECL (Pierce) or incubation with 0.1% (w/v) 4-chloro-1-naphthol (Sigma) and 0.5% (v/v) hydrogen peroxide in TBS. GFP in load and eluate fractions was detected as a single band of ∼28 kDa.

Target protein concentration and purity were measured from various stages of the purification by GFP ELISA (Cell Biolabs) and Experion Pro260 microfluidic capillary electrophoresis (Bio-Rad), respectively, according to the manufacturers’ instructions. The GFP ELISA kit has a reported detection sensitivity limit of 30 pg/ml, and samples were diluted to be within the dynamic range of the assay parameters and purified GFP protein standards. Microfluidic capillary electrophoresis permits electropherogram quantification of total protein content separated by molecular weight and has a reported detection limit of 2.5 ng/ml. Final GFP concentrations for all samples were normalized to original HIC load volume. Post-run eluate analysis data were compared to in-line spectrophotometric elution profiles in order to determine the extent to which in-line A_395_ measurements could be used as a suitable indicator of GFP for subsequent purifications.

## Results and Discussion

### HIC Column Scouting and Capture of GFP from Bacterial Lysate

**Figure 1:**
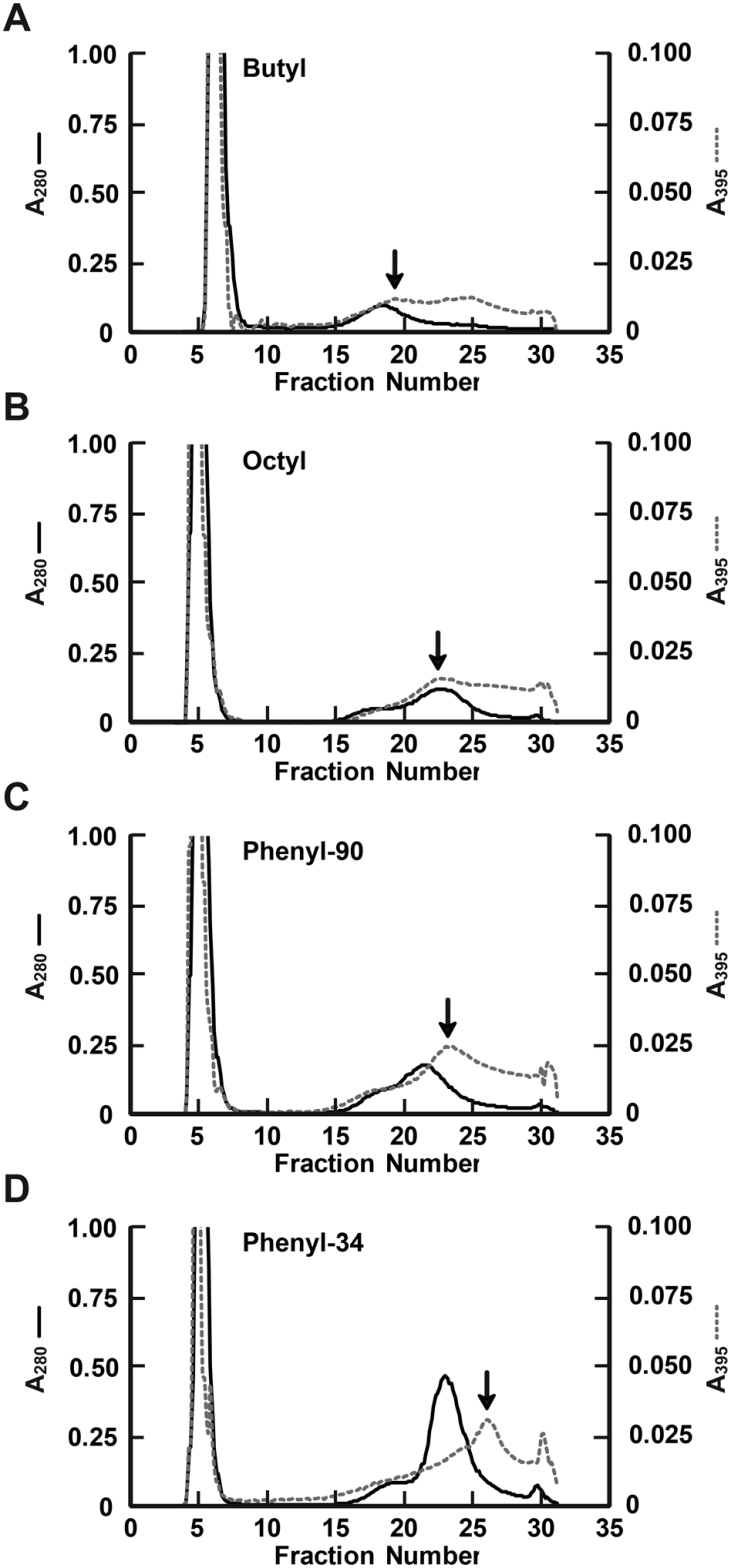
In-line spectrophotometric detection of total protein and GFP elution during HIC media column scouting. The amount of total protein and GFP present in each fraction was simultaneously estimated by assaying in-line spectrophotometric absorbance of light at 280 nm (*solid line*, *A_280_*) and 395 nm (*dashed line*, *A_395_*), respectively. Seven different HIC media were scouted for target protein (i.e., GFP) separation characteristics, and A_280_ and A_395_ elution profiles of the butyl (*A*), octyl (*B*), phenyl-90 (*C*), and phenyl-34 (*D*) scouting runs are shown. The y-axes of each panel are drawn to identical scales in order to allow a direct visual comparison of representative peak heights between scouting runs. The *arrow* in each panel identifies the retained eluate fraction that displayed the greatest A_395_ absorption, a presumed indicator of GFP concentration.

In-line spectrometric analysis following HIC column scouting indicated notable differences in elution profiles of total protein and GFP among the seven HIC media initially tested. Because there is limited predictability of target protein biding affinity for individual HIC media and HIC column scouting of untagged GFP captured from crude bacterial lysate has not been previously reported, these results could not have not been predicted without experimentation. The void volume for all media ranged from fractions 4–7 and retained proteins eluted from approximately fractions 15–32. The A_280_ and A_395_ in-line elution profiles for total protein and GFP of the four media with the most distinct chromatographic properties–butyl, octyl, phenyl-90, and phenyl-34–are presented in Figure 1. Butyl, octyl, and phenyl media all eluted GFP over an equivalent number of fractions; however, the butyl and octyl spectrophotometric profiles lack the well-defined A_395_ absorbance peak, presumably indicative of GFP, that was observed with the phenyl-90 and phenyl-34 media. The diameter of the matrix beads notably impacted separation, as the 34 µm-diameter phenyl media produced a more distinct A_395_ peak than the 90 µm phenyl media. The phenyl-34 medium produced the most distinct A_395_ peak of all HIC media scouted. GFP eluted earliest with the butyl-based media and was retained the longest with the phenyl media.

It should also be noted that, although A_395_ is a peak GFP absorption wavelength and approximate indicator of GFP presence, other proteins, including those with heme groups and conjugated ring systems also absorb light at 395 nm [19], [20]. As a consequence, A_395_ is a relative, but not absolute, indicator of GFP content. Prior to concluding that the presumptive GFP elution profile generated by A395 is fully indicative of target protein concentration, additional analyses of the fractionated (e.g., post-run fluorimetry, Coomassie staining, western blotting, or ELISA) must be performed.

The A_280_ profiles indicated that the butyl and octyl media retained less total protein, and that the greatest amount of retention was observed with the phenyl-34 medium. For all columns tested, a single main peak of total protein (A_280_) eluted at or before the maximum A_395_ elution peak (Figure 1). The octyl medium produced the least resolved separation between A_395_ and A_280_ chromatographic traces, showing peak absorptions of both wavelengths in the same fraction. The best spectrophotometric separation of A_395_ and A_280_ elution peaks occurred using the phenyl-34 medium. Separation between A_395_ and A_280_ provided a preliminary indication that GFP was at least partially isolated from the remainder of bacterial lysate protein. The observed binding capacity and A_395_-A_280_ peak separation of the phenyl-34 column can likely be attributed to a combination of its high degree of ligand hydrophobicity, increased ligand density, and small matrix bead size. While phenyl-34 produced the best target protein capture for GFP under the experimental conditions tested, other HIC-compatible proteins, particularly those with a lower degree of hydrophobic interaction, may preferentially resolve using a different HIC buffer system or weaker hydrophobic ligand, such as butyl- or octyl-based HIC media [21], [22]. Decreasing the chromatographic flow rate, sample concentration, or sample load volume are also commonly used HIC modifications to improve separation [1], [3], [5].

**Figure 2:**
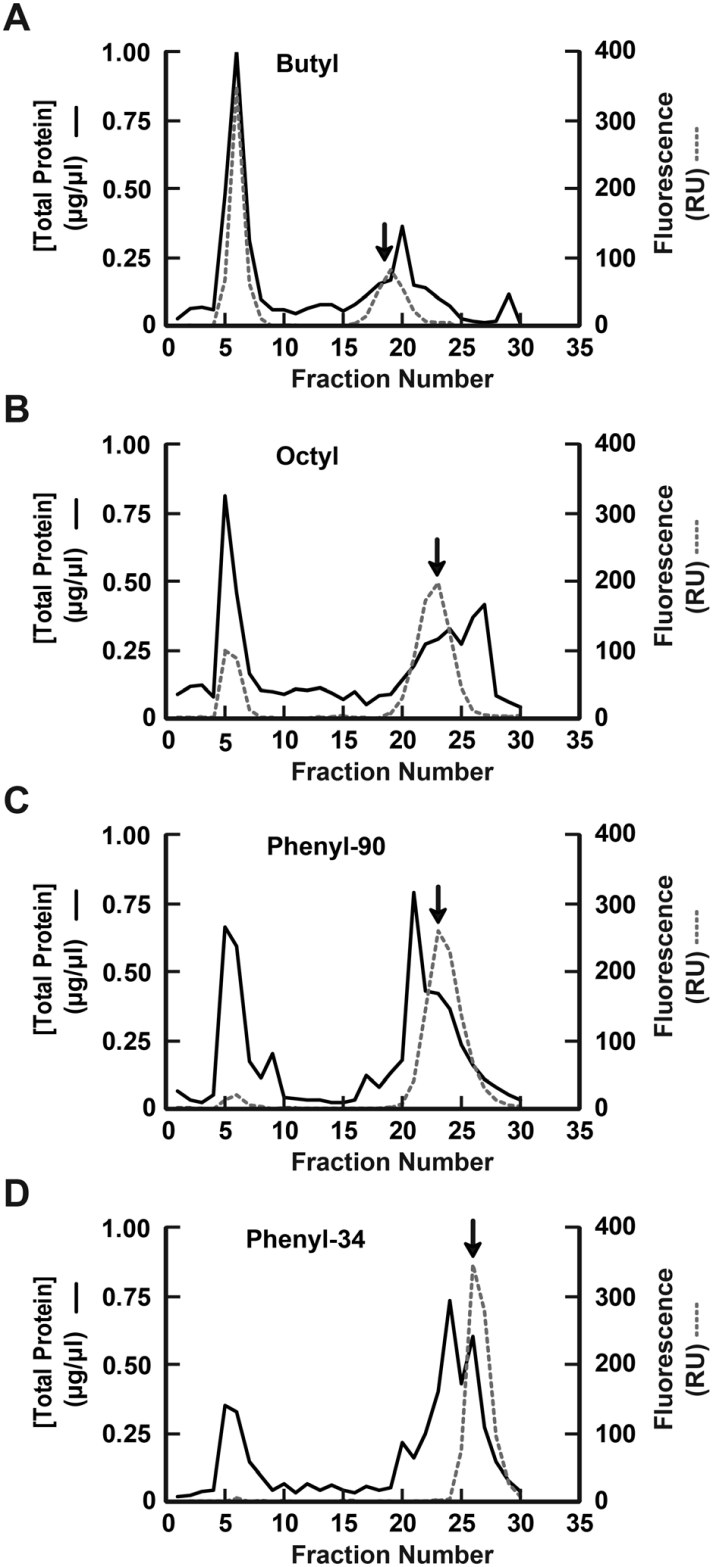
Post-run eluate analysis detection of total protein and GFP elution profiles from HIC column scouting. Following fractionation of GFP-containing lysate using the seven HIC media described above, the amount of total protein and GFP present in each eluate fraction were estimated by Bradford assay (*solid line*) and fluorimetry (*dashed line*), respectively. Total protein and GFP elution profiles of the butyl (*A*), octyl (*B*), phenyl-90 (*C*), and phenyl-34 (*D*) scouting runs are shown. Y-axes of each panel are drawn to identical scales for ease of direct visual comparison. Fluorescence intensities are expressed in relative units (*RU*). *Arrows* indicate the fraction from each scouting run containing the highest concentration of retained GFP, as predicted by post-run fluorimetry.

In order to confirm in-line A_395_ and A_280_ spectrophotometry data, elution profiles of total protein and GFP were generated from post-run analysis of the fractionated eluate using Bradford assay and fluorimetry detection, respectively (Figure 2). Based on the collected fluorimetry data, the majority of GFP loaded onto the butyl column seemingly failed to adhere to the column under the HIC conditions tested, and the relatively small amount of GFP that was retained eluted with the bulk of total protein and over a span of 6–7 fractions. The octyl- and phenyl-based media resulted in better GFP separation from total protein, and more GFP was retained with the phenyl-90 and phenyl-34 media than the octyl. Of the two phenyl-based media, phenyl-34 produced a more concentrated GFP elution peak of 4–5 fractions, compared to the 8 fractions of the phenyl-90. No fluorescence peak was observed in the void volume of the phenyl-34 column and minimal fluorescence was detected in the phenyl-90 void volume, indicating extensive GFP adsorption to these columns (Figure 2).

The post-run eluate analysis techniques shown in Figure 2 demonstrate greater differences among media than would have been predicted based on the in-line A_280_ and A_395_ spectrophotometry data presented in Figure 1. GFP has an absorbance (i.e., excitation) maximum at 395 nm and emission (i.e., fluorescence) maximum at 509 nm. A notable level of A_395_ detected in bacterial cell lysate was not related to GFP, specifically in the washout and other fractions containing high concentrations of bacterial lysate protein.

For each medium tested, the presumptive eluate fraction containing the greatest amount of GFP was identical between post-run and in-line detection methods (indicated by arrows, Figures 1–2); however, the shape of the GFP elution peak was notably sharper for all media as determined by post-run fluorescence. Detecting green light fluorescence in fractionated eluate (dotted lines, Figure 2) is a more unique indicator of GFP than 395 nm light absorption (dotted lines, Figure 1), yet the two detection methods are useful in combination to provide a more detailed analysis of HIC elution profiles.

Differences between the in-line and post-run analysis data may have resulted from sensitivities of the detection methods, pooling of the eluate fractions versus continual in-line monitoring, and the specificity of each method for recognizing only GFP. The difference between in-line absorbance and post-run fluorescence is certainly notable, indicating the importance of utilizing a consistent approach and recognizing that A_395_ is indicative of, but not specific to, GFP. Utilizing an in-line 509 nm fluorescence detector would presumably have resulted in identification of GFP peaks more similar to those obtained by post-run fluorescence profiles rather than in-line A_395_. The selection of in-line or post-run analysis methods may be based on user preference or available instrumentation capabilities. Despite the differences in presumptive GFP elution profiles, both methods accurately identified the same postulated GFP elution peak, and either technique may be used in conjunction with Coomassie staining and western blot analysis in selecting an optimal HIC medium and desired elution fractions for target protein capture.

**Figure 3:**
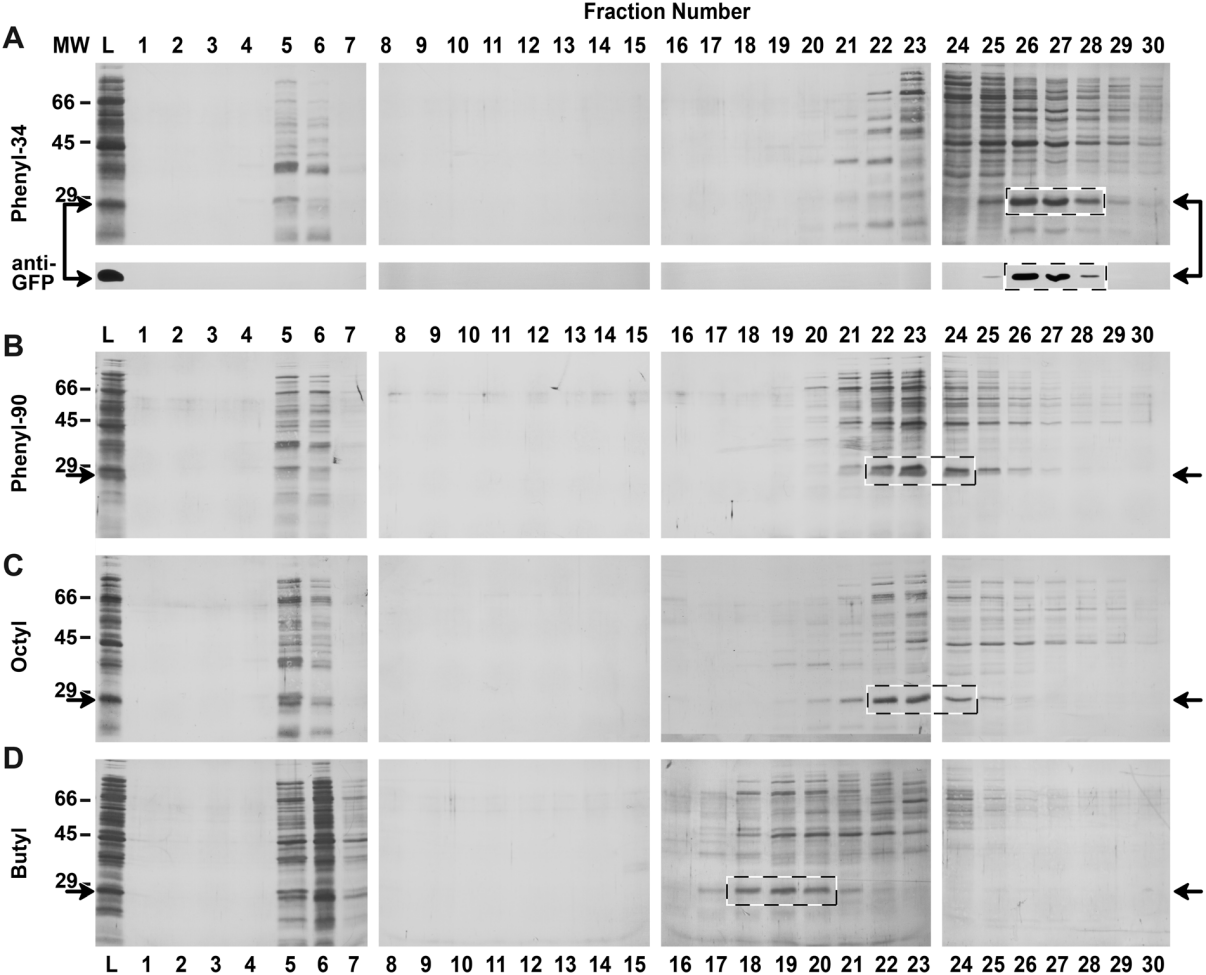
Coomassie staining and western blot analysis of total protein and GFP elution from HIC column scouting. Aliquots from each scouting run eluate fraction were electrophoresed under denaturing conditions, electrotransferred, and Coomassie stained. Coomassie stained membranes of phenyl-34 (*A*), phenyl-90 (*B*), octyl (*C*), and butyl (*D*) HIC eluate fractions are shown as representative examples of the seven HIC media scouted. Duplicate SDS-PAGE membranes for all scouting runs were western blotted using *anti-GFP* antibody. The western blot of the phenyl-34 fractions is shown below the corresponding phenyl-34 Coomassie stained membranes and is representative of the blotting results obtained for each media tested (data not shown). The transit time (i.e., washout) of the HIC chromatography system corresponds to the first 5–7 fractions of each scouting run. For the retained protein on each column scouted, western blotting detected a single ∼28 kDa band elution peak, and the signal intensity coincided with the most prominent ∼28 kDa band observed by Coomassie staining (*dashed boxes*) and the single fluorimetry peak observed in Figure 2. Membranes for the four scouting runs are arranged identically by fraction number to enable direct visual comparisons of protein staining and elution profiles. *Arrows* indicate the anticipated location of GFP in each membrane, as determined by molecular weight and western blot. *L*, GFP-containing bacterial lysate loaded onto the HIC columns; *MW*, molecular weight of protein standards (kDa).

The eluate fractions for the four representative HIC columns–butyl, octyl, phenyl-90, and phenyl-34–were resolved by SDS-PAGE and Coomassie stained for total protein (Figure 3), and the presence or absence of GFP in each fraction was confirmed by western blot. GFP resolves as a ∼28 kDa band under denaturing conditions (Figure 3, indicated by arrows), and loading an equal volume of all fractions from the columns enabled comparison of relative GFP retention and elution profiles between media. It is notable that GFP is not the only 25–30 kDa protein present in the bacterial cell lysate loaded onto the HIC columns, thus western blotting is beneficial for confirming GFP protein identity.

The relative separation of GFP from cytosolic proteins shown in Figure 3 mirrors the post-run fluorimetry and Bradford profiles shown in Figure 2. From these data, we concluded that the phenyl-34 medium (Figure 3A) provided the greatest amount of GFP retention and separation, and was thus identified as the preferred HIC column to be used for the initial capture step of sequential HIC-SEC protein purification. Using this column, retained GFP eluted later than the major peak of bacterial lysate proteins and no GFP was detected in the void volume (fractions 5–6), as determined by western blot. Fractions from the butyl-based purification (Figure 3D) depicted a dense 28 kDa GFP band in the void volume (fractions 5–7) and only a faint band in later fractions, which corresponded to elution of the retained GFP. These data indicate poor GFP binding capacity for butyl-based media under the HIC conditions tested and are consistent with conclusions drawn from the post-run fluorescence detection of GFP shown in Figure 2A. Octyl and phenyl-90 columns (Figure 3 B–C) provided less adequate target protein separation and less total protein retention than obtained with phenyl-34.

Modifications to the HIC protocol, such as altering pH, elution buffer, or ionic strength, would likely alter the observed chromatographic separation profiles and may result in selection of a different optimal HIC medium. A common HIC method development approach is to first scout for a suitable medium for target protein capture, followed by altering HIC conditions if improved separation is necessary [2], [3], [4]. The phenyl-34 column appeared to provide adequate GFP capture, such that the protein could be purified through subsequent polishing steps without the need for scouting additional HIC parameters.

### Polishing of Captured GFP by Size Exclusion Chromatography

GFP was next separated from bacterial lysate proteins that co-eluted during the initial HIC capture using SEC. Eluate fractions from the HIC phenyl-34 scouting run containing retained GFP (fractions 26–28, boxed region in Figure 3A) were pooled, concentrated to 1 ml, and loaded onto a Superdex 200 SEC. A relatively narrow range of three HIC fractions was selected in order to minimize contamination with other bacterial lysate proteins. The pooled fractions were filtered through the SEC column at a constant flow rate under isocratic buffer conditions.

**Figure 4:**
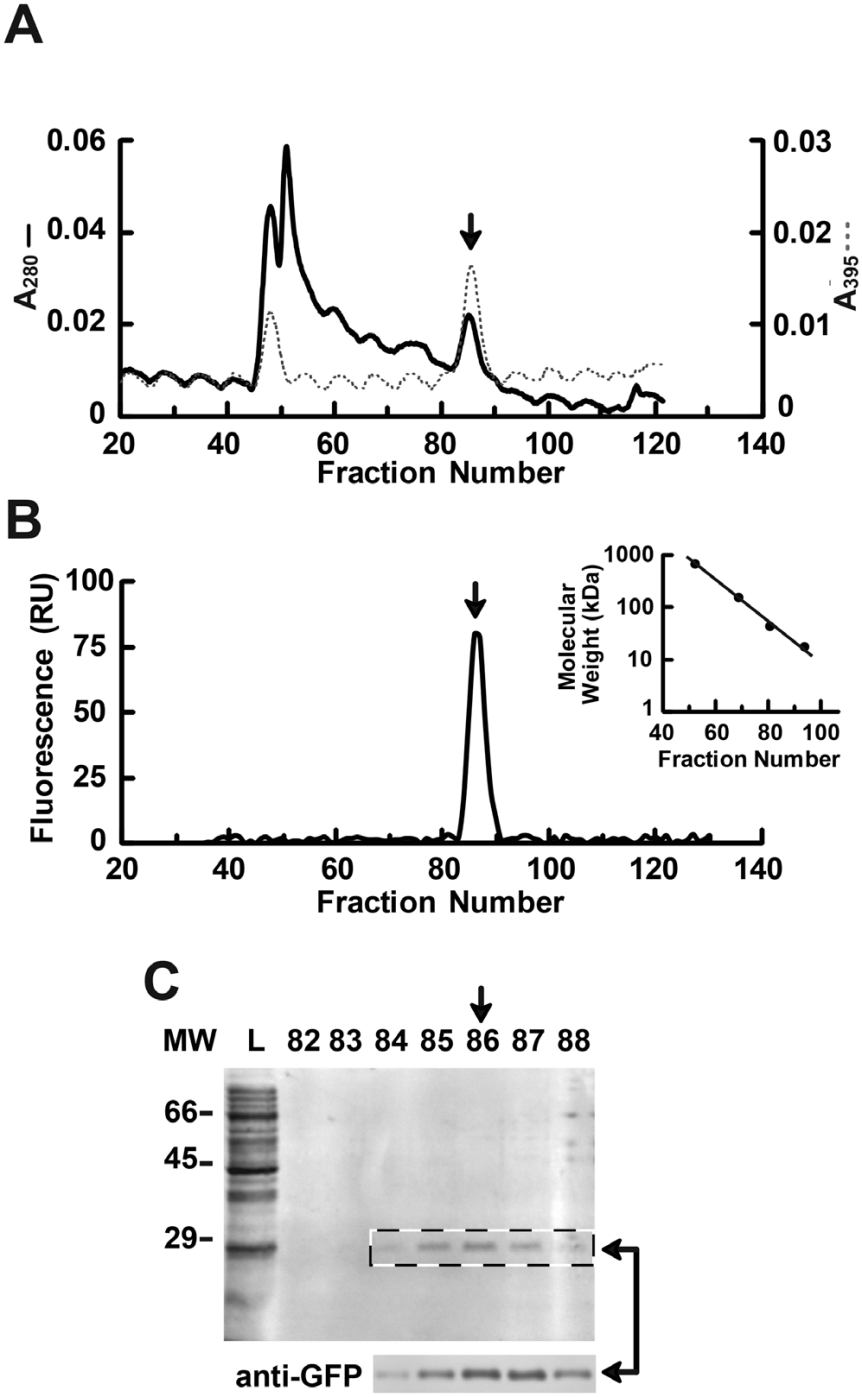
Size exclusion chromatography elution profile of pooled GFP-containing HIC eluate. Eluate fractions containing GFP from the phenyl-34 HIC scouting run (*dashed box,* *Figure 3A*) were pooled, concentrated, and loaded onto an SEC column. Total protein and GFP were monitored during SEC elution using in-line and post-run detection methods, as described above. *Vertical arrows* indicate the major GFP peak observed using each detection method. *A*, In-line spectrophotometric indication of total protein (A_280_, *black line*) and GFP (A_395_, *grey line*). The episodic baseline fluctuation observed in the A_280_ and A_395_ tracings is a detection artifact. *B*, Post-run fluorometric detection of GFP, expressed in relative units (*RU*). The *inset* indicates elution of gel filtration molecular weight standards on the SEC column used to calculate the size of the eluted protein peak. *C*, Coomassie stain (*above*) and western blot (*below*) of SEC eluate fractions surrounding the GFP elution peak observed in A *and* B. The anti-GFP western blot for all SEC fractions (1–122) produced only a singe band, which corresponded in size and intensity to the ∼28 kDa protein detected by Coomassie stain in fractions 84–88 (*dashed box*), indicated by *horizontal arrows*. Fractions 84–88 were pooled and concentrated for subsequent analysis. *L*, pooled GFP-containing phenyl-34 HIC eluate loaded onto the SEC column; *MW*, molecular weight of protein standards (kDa).

The in-line analysis profile of the SEC eluate identified two separate peaks of absorbance at 395 nm (Figure 4A). Relative positioning of A_395_ and A_280_ and SEC molecular weight standards indicated that the first peak (fractions 45–51) correlated to the void volume and very high molecular weight proteins (>600 kDa). The second in-line A_395_ peak (fractions 84–88, marked with an arrow), corresponded to a protein with a molecular weight of ∼25–35 kDa. Post-run fluorimetry analysis of the fractionated eluate identified a single peak of 509 nm green light fluorescence, which also occured in fractions 84–88 (Figure 4B). Coomassie staining and western blotting, considered in conjunction with SEC molecular weight calculations, confirmed the isolated protein detected in these SEC fractions to be monomeric GFP (Figure 4C). As such, post-run analysis of the SEC eluate identified the major GFP peak predicted by in-line A_395_ detection. No GFP was present in the SEC void volume, as determined by fluorimetry and western blotting, despite detection of absorbance at 395 nm (cf., Figures 4A and 4B). The presence of an A_395_ elution peak corresponding to the SEC void volume, presumably from bacterial lysate proteins unrelated to GFP, was consistent with the A_395_ data observed for the phenyl-34 HIC scouting run (cf., Figures 1D and 2D).

**Figure 5:**
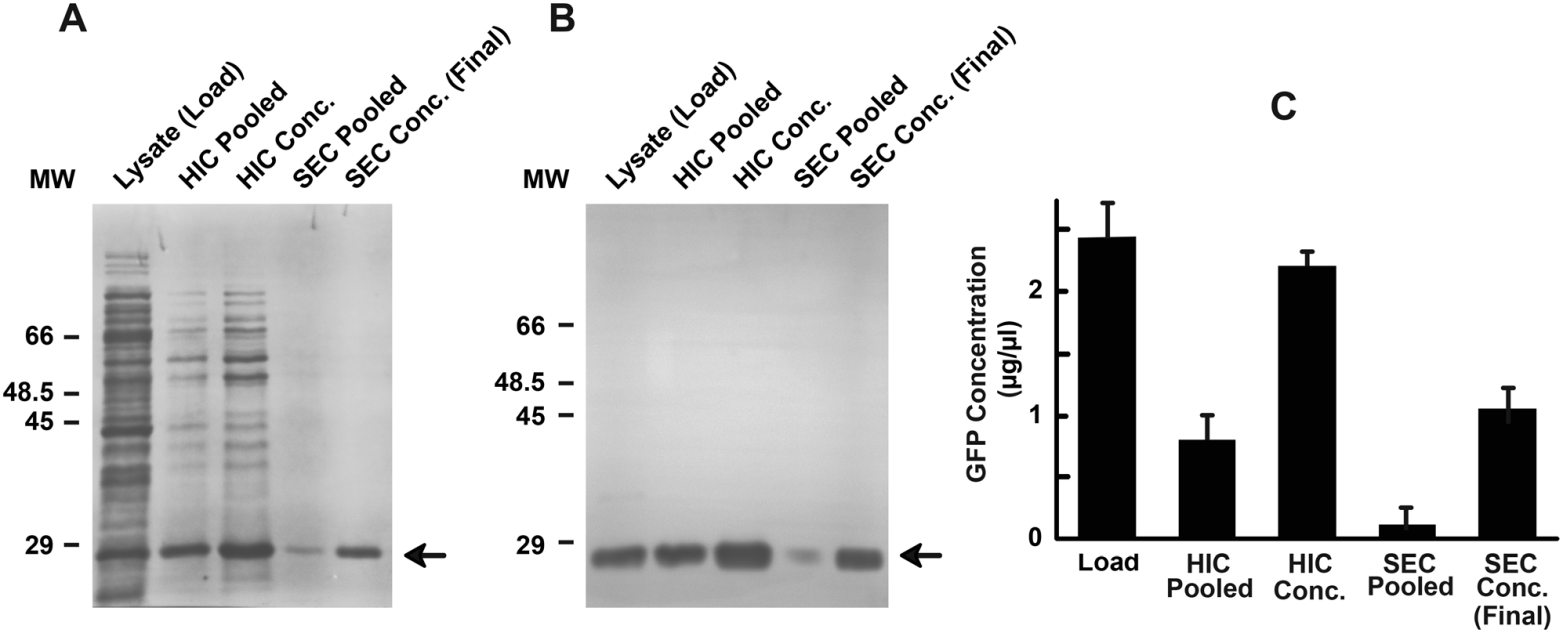
Comparison of total protein and GFP across the two-step purification process. GFP was sequentially purified by HIC and SEC, as described above, and aliquots from each step were compared side-by-side using Coomassie stain (*A*), western blot (*B*), and ELISA (*C*) to assess changes in GFP purity and concentration. GFP-containing bacterial lysate (*Lysate*) was loaded onto the phenyl-34 column and fractioned using HIC. The contiguous HIC fractions containing high levels of retained GFP were collected (*HIC Pooled*) and then concentrated (*HIC Conc.*). The concentrated GFP-containing HIC eluate was further separated from contaminant proteins by SEC, and SEC fractions containing retained GFP were collected (*SEC Pooled*) and then concentrated (*SEC Conc.*) to yield the final, purified GFP product. An equal volume of *HIC Pooled*, *HIC Conc.*, *SEC Pooled*, and *SEC Conc.* was electrophoresed in in order to permit a direct visual comparison of protein concentration across the purification procedure. The volume of *Lysate* electrophoresed was half the volume of the other samples in order to prevent overloading of the SDS-PAGE gel. The western blot shown in *B* was probed using anti-GFP antibody, and the full-length membrane is displayed in order to demonstrate the high degree of antibody specificity. The ELISA in *C* quantifies the concentration of GFP in each step of the purification. Samples were assayed in triplicate and bars represent the average concentration ± SEM.

The purity, abundance, and yield of GFP following sequential HIC and SEC fractionation were determined by Coomassie stain, western blot, and ELISA, respectively (Figure 5). Pooled HIC and SEC fractions containing GFP were concentrated back to the 1 ml original load volume in order to provide a more direct visual comparison of protein concentration and activity. Total protein composition of the initial GFP-containing bacterial lysate, pooled and concentrated HIC fractions, and pooled and concentrated SEC fractions were visualized by Coomassie stain (Figure 5A). The initial lysate loaded onto the HIC column contained many dense high molecular weight protein bands, which were subsequently removed by HIC and SEC. The concentrated SEC fractions exhibited one clear band of ∼28 kDa, corresponding to highly purified GFP. Western blotting of duplicate PVDF membranes demonstrated both anti-GFP antibody specificity and relative GFP concentration of the final protein product (Figure 5B). ELISA analysis showed the bacterial lysate loaded onto the HIC possessed 2.4 µg/µl GFP, and the final, concentrated sample at the conclusion of the purification contained 1.13 µg/µl GFP, which was 47% of the initial load concentration (Figure 5C). A relatively small number of fractions were pooled from both HIC and SEC elutions for subsequent purification steps and, even more notably, the large bed volume of the SEC column presumably contributed to decreasing the target protein yield below that which could have been optimally achieved. Utilization of a shallower HIC salt gradient and a much smaller SEC column both have the potential to increase target protein yield without sacrificing protein purity. Maximizing yield is particularly important for purifications involving target proteins that are available in limited quantities, such as poorly expressed or endogenous proteins; however, purifications of readily expressed recombinant target proteins, such as GFP, generally require substantially less stringent yield optimization.

**Figure 6:**
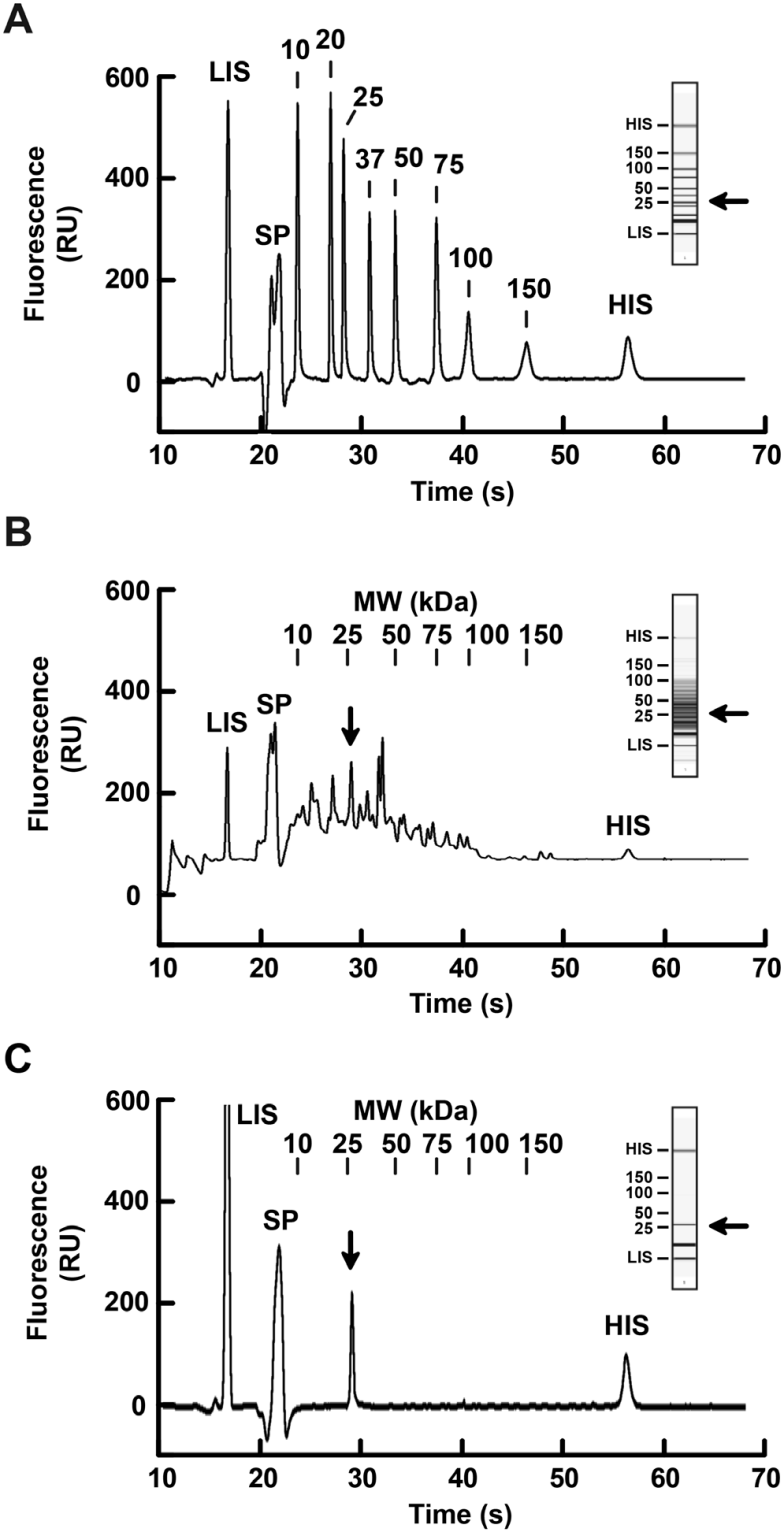
Final GFP protein purity, concentration, and comparison to crude lysate using microfluidic capillary electrophoresis. *A*, Electropherogram of 10–150 kDa molecular weight standards. The *numbers* indicate the molecular weight of each standard protein peak expressed in kDa. These numbers are aligned at the top of panels B and C and indicate molecular weights observed in those samples as well. *B*, Electropherogram of the GFP-containing bacterial lysate, which served as the initial sample load used for HIC column scouting. *C,* Electropherogram of the concentrated GFP-containing SEC fractions 84–88, the final product of the purification scheme. Fluorescence intensity expressed in relative units (*RU*). *Panel insets*, Virtual gels generated from electropherogram data. *Arrows*, theoretical molecular weight of GFP. *LIS*, low MW internal standard; *HIS*, high MW internal standard; *SP*, system peaks generated by the electrophoresis system unrelated to the sample being assayed.

Microfluidic capillary electrophoresis was used to quantify GFP purity, beyond what could be detected using Coomassie staining and densitometry calculations (Figure 6). Electropherograms mirrored Coomassie stained gels, indicating protein peaks of varying molecular weights were present in the GFP-containing bacterial lysate initially loaded onto the HIC columns for scouting (Figure 6B). The 28.5 kDa peak detected in this lysate was calculated as accounting for 8.8% of total protein. As shown in Figure 6C, a single, 28.5 kDa protein peak was observed in the final concentrated sample and no additional protein contaminants were detected in the final product. Using a conservative estimate and factoring in the instrumentation detections limits, the calculated GFP purity is >98%. The level of methodological detail presented here and characterization of purified GFP protein product may be particularly instructive to the investigator nascent to HIC methods development or in educational settings in which GFP is a commonly used model protein and various approaches to chromatographic protein purification are discussed.

Taken together, these results have shown that semi-automated HIC column scouting allowed for selection of a HIC medium suitable for two-step purification of recombinant GFP. We identified a phenyl ligand coupled to a 34 µm resin as the preferred HIC medium, which allowed for ample second-step polishing via high resolution preparative SEC. Column scouting coupled with in-line spectrophotometric analysis enabled relatively rapid selection of a preferred chromatographic medium; however, the presumptive GFP elution profile required verification using post-run detection methods that are more specific indicators of target protein presence and concentration.

Although rudimentary HIC-based protein purifications may be conducted using relatively basic instrumentation, such as a single bench top gravity flow column, modern FPLC systems enable increased protein analysis capability and purification efficiency. The dynamic sample loop injection, sequential column selection, multi-wavelength analysis, and split fraction eluate collection increased the functionality of the system and reproducibility of the experimental approach. From an instructional perspective, it is of note to compare similarities and differences associated with target protein purification using a variety of instrumentation and approaches [10], [23]. While empirical determination of optimal HIC media and buffer conditions are commonly required for each new HIC-based protein purification, the approach presented here could serve as an initial template to be applied to other dual HIC-SEC target protein purification strategies.

## Supporting Information

Figure S1 Schematic illustration of the medium-pressure liquid chromatography system used in this study. The system includes *buffer maximizer valves*, dual-piston *pump workstation*, and in-line *mixer*, which allow for the coordinated blending of four stock buffers in order to control the pH and ionic strength of ammonium phosphate present in the mobile phase. Crude lysate is loaded into a *dynamic sample loop* through the use of a low-pressure peristaltic *sample loop loading pump*, and an *air sensor* protects air bubbles from being drawn into the system. Following loading of lysate into the dynamic sample loop, the internal flow path of the sample loading valve switches to allow injection of the sample from the dynamic sample loop onto the HIC columns. A pair of *column selection valves* located immediately upstream and downstream of the *HIC columns* to be scouted regulate through which column buffer and sample flow. The column selection valves function in parallel to facilitate movement of the mobile phase through a single column at any one time. For subsequent chromatography runs (e.g., SEC purification of the HIC eluate), one HIC column and stock buffer were replaced with an SEC column and HE buffer (not shown). In-line analysis components downstream of the HIC columns and selective valves, including a *multiple wavelength detector*, single-wavelength *UV/Vis detector*, *conductivity monitor*, and *pH monitor*, permit real-time monitoring of the column eluate as it is being fractionated and collected. A 40 psi *backpressure regulator* maintains unidirectional mobile phase flow and is intentionally placed upstream of detectors that require a low-pressure environment. An *alternating valve* controlled by an *auxiliary pump* unit diverts eluate between a pair of *fraction collectors*, such that 90% of the eluate is collected in 12 ml test tubes and 10% is collected in 96 well plates. Split fraction collection increases the ease of post-run eluate analysis. Controllable valves are identified with their names written in *purple*.
(TIF)
