# Supplementary figures and images for: Semi-Automated Hydrophobic Interaction Chromatography Column Scouting Used in the Two-Step Purification of Recombinant Green Fluorescent Protein
Source: PLoS One. 2014 Sep 25;9(9):e108611. doi: 10.1371/journal.pone.0108611 (PMC4177899; doi:10.1371/journal.pone.0108611)

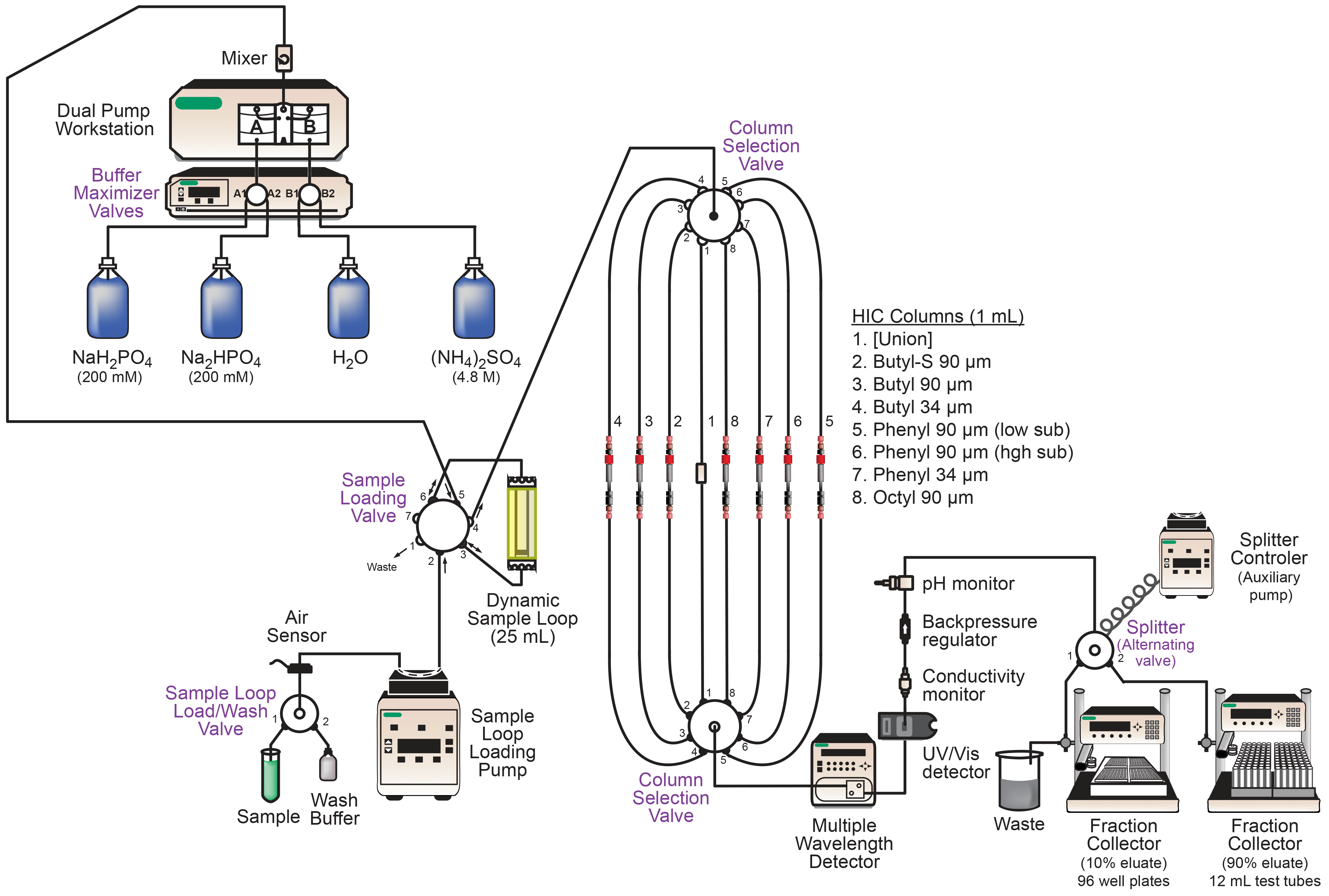

Supplement: Figure S1 — Schematic illustration of the medium-pressure liquid chromatography system used in this study. The system includes buffer maximizer valves, dual-piston pump workstation, and in-line mixer, which allow for the coordinated blending of four stock buffers in order to control the pH and ionic strength of ammonium phosphate present in the mobile phase. Crude lysate is loaded into a dynamic sample loop through the use of a low-pressure peristaltic sample loop loading pump, and an air sensor protects air bubbles from being drawn into the system. Following loading of lysate into the dynamic sample loop, the internal flow path of the sample loading valve switches to allow injection of the sample from the dynamic sample loop onto the HIC columns. A pair of column selection valves located immediately upstream and downstream of the HIC columns to be scouted regulate through which column buffer and sample flow. The column selection valves function in parallel to facilitate movement of the mobile phase through a single column at any one time. For subsequent chromatography runs (e.g., SEC purification of the HIC eluate), one HIC column and stock buffer were replaced with an SEC column and HE buffer (not shown). In-line analysis components downstream of the HIC columns and selective valves, including a multiple wavelength detector, single-wavelength UV/Vis detector, conductivity monitor, and pH monitor, permit real-time monitoring of the column eluate as it is being fractionated and collected. A 40 psi backpressure regulator maintains unidirectional mobile phase flow and is intentionally placed upstream of detectors that require a low-pressure environment. An alternating valve controlled by an auxiliary pump unit diverts eluate between a pair of fraction collectors, such that 90% of the eluate is collected in 12 ml test tubes and 10% is collected in 96 well plates. Split fraction collection increases the ease of post-run eluate analysis. Controllable valves are identified with their [file pone.0108611.s001.tif]
